# Prenatal exposure to concentrated ambient PM_2.5_ results in spatial memory defects regulated by DNA methylation in male mice offspring

**DOI:** 10.1007/s11356-022-24663-5

**Published:** 2022-12-17

**Authors:** Yingying Yang, Tingting Yang, Ji Zhou, Zhijuan Cao, Zehuan Liao, Yan Zhao, Xiujuan Su, Jia He, Jing Hua

**Affiliations:** 1 Department of Women and Children’s Health Care, Shanghai Key Laboratory of Maternal Fetal Medicine, Shanghai First Maternity and Infant Hospital, School of Medicine, Tongji University, Shanghai, China; 2 Department of Medical Epidemiology and Biostatistics, Karolinska Institutet, Stockholm, Sweden; 3 Department of Social Medicine, School of Public Health, Fudan University, Shanghai, China; 4 Shanghai Key Laboratory of Meteorology and Health, Shanghai Meteorological Bureau, Shanghai, China; 5 Shanghai Typhoon Institute, CMA, Shanghai, China; 6 Department of Atmospheric and Oceanic Sciences, & Institute of Atmospheric Sciences, Fudan University, Shanghai, China; 7 School of Biological Sciences, Nanyang Technological University, Singapore, Singapore; 8 Department of Microbiology, Tumor and Cell Biology (MTC), Karolinska Institutet, Stockholm, Sweden; 9 School of Medicine, Tongji University, Shanghai, China

**Keywords:** Prenatal exposure, Concentrated ambient PM_2.5_, Mice offspring, Spatial memory defects, Interleukin 6 (IL-6), DNA methylation

## Abstract

Ambient fine particulate matter (PM_2.5_) exposures during pregnancy could lead to adverse birth outcomes, including neurobehavioral development defects. However, limited studies explored the effects and potential epigenetic mechanisms of maternal PM_2.5_ exposure on offspring spatial memory defects. This study aims to explore the effects and underlying epigenetic mechanisms of maternal concentrated ambient PM_2.5_ exposure in male mice offspring with spatial memory defects. Pregnant female C57BL/6 mice were exposed daily to concentrated ambient PM_2.5_ (CAP) or filtered air (FA) throughout gestation, with the concentration of particulates (102.99 ± 78.74 μg/m^3^) and (2.78 ± 1.19 μg/m^3^), respectively. Adult male mice offspring were subsequently assessed for spatial learning and memory ability using Morris Water Maze tests and locomotor activities in open field tests. The hippocampus of the male mice offspring was harvested to test mRNA expression and DNA methylation. Results from the probe test of Morris Water Maze showed that the mice offspring in the CAP group had shorter swimming distance travelled in the target quadrant, shorter duration in the target quadrant, and less number of entries into the target quadrant (*p* < 0.05), suggesting spatial memory impairments. The acquisition trials of Morris Water Maze did not show a significant difference in learning ability between the groups. The mRNA level of interleukin 6 (IL-6) in the CAP group hippocampus (10.80 ± 7.03) increased significantly compared to the FA group (1.08 ± 0.43). Interestingly, the methylation levels of the CpG sites in the IL-6 promoter region declined significantly in the CAP group, (5.66 ± 0.83)% vs. (4.79 ± 0.48)%. Prenatal exposure to concentrated ambient PM_2.5_ induced long-lasting spatial memory defects in male mice offspring. The underlying biological mechanism might be mediated by an inflammatory reaction which is regulated by DNA methylation.

**Supplementary Information:**

The online version contains supplementary material available at 10.1007/s11356-022-24663-5.

## Introduction

Air pollution exposure is a worldwide environmental problem, which is one of the main contributors to the global disease burden (Collaborators 2018). With rapid industrial development and overpopulation, air pollution was one of the serious problems in many developing countries, including China. Air pollution contained mixtures of solid particles and liquid droplets found in the air. Among the key air pollutants, fine particulate matter (PM_2.5_), with aerodynamic diameters equal to or less than 2.5 μm, could be inhaled and cause adverse health effects in many different aspects. The temporary effects caused by short-term PM_2.5_ exposure ranged from simple discomfort (wheezing, coughing, etc.) to more serious states (asthma, pneumonia, lung and heart problems, etc.). The long-term PM_2.5_ exposure was harmful to the neurological, reproductive, respiratory systems, and cardiovascular and respiratory systems and causes cancer (Manisalidis et al. 2020). Studies have also suggested that perinatal exposure to ambient PM_2.5_ could affect placental development and function (Zanini et al. 2020), thus resulting in short-term adverse outcomes in offspring, including declined birth weight, intrauterine developmental restriction, and premature delivery (Dadvand et al. 2013; Hjortebjerg et al. 2016; Trasande et al. 2016).

The studies on the health impacts of air pollution have long been focused on cardiovascular and pulmonary systems in the past decades. However, an increasing amount of evidence demonstrated that the central nervous system can also be deleterious by air pollution exposure. Neurodevelopment began in the early life within the embryo during pregnancy, which was an important and sensitive time window for the whole lifespan. The development of the neural system contained some complex and critical developmental processes, including proliferation, migration, differentiation, synaptogenesis, myelination, and apoptosis. (Rice and Barone 2000). These processes continued to develop in the postnatal years. As the sensitive period, the nervous development in the embryo and fetus during pregnancy was vulnerable to environmental insults, including air pollutants (Shang et al. 2020), which may have consequences for the nervous system development in offspring.

The developmental origins of health and disease (DOHaD) hypothesis demonstrated that the adverse exposures in early life could permanently shape the molecular programming and contributed to later disease predisposition (Godfrey and Barker 2001, Barker 1997). Multiple studies demonstrated that a high level of PM_2.5_ exposure in utero and early life was associated with neurodevelopmental disorders, long-lasting behavioral alterations, and cognitive defects in offspring (Patten et al. 2020; Buoli et al. 2018). Epidemiological studies suggested that a higher level of prenatal PM_2.5_ exposure was associated with the declined function of memory and attention domains (Chiu et al. 2016). Animal studies demonstrated exposure to traffic-related air pollution from approximately gestational day 14 through postnatal days 41–51 affected neurodevelopment in rat offspring experiments (Patten et al. 2020). High dose of PM_2.5_ exposure through intranasal instillation during postnatal days 3–15 impaired spatial learning and memory abilities in immature rats (Liu et al. 2019). However, some areas of the central nervous system, such as the forebrain, midbrain, and hindbrain began to form with the neurogenesis and migration of cells earlier than the second week of gestation in rodents (since gestational day 7 in mouse, and 9.5 days in rats) (Rice and Barone 2000). The PM_2.5_ exposure animal models in previous studies were unlike real-world exposure (Liu et al. 2019). In addition, both population (Lertxundi et al. 2019; Chiu et al. 2016) and experimental studies (Allen et al. 2014; Bolton et al. 2017) demonstrated sex-dependent neuropsychological effects due to prenatal air pollution exposure, which is especially detrimental for males.

Previous experimental studies using animal models tried to explore the cognitive consequences of air pollution exposure. However, the neurotoxicological mechanism underlying these associations had yet to be clarified. Furthermore, the biological mechanisms were likely to differ depending upon the exposure period underway the brain development period, and the components of the air pollution exposure itself (Allen et al. 2017a). The potential contributory mechanism of PM_2.5_ exposure affecting cognition and behavior included oxidative stress/inflammation (Zanchi et al. 2010; Gerlofs-Nijland et al. 2010), DNA methylation (Boda et al. 2020), and structural synaptic plasticity disruption (Liu et al. 2019). DNA methylation, a characterized epigenetic modification, might be one of the potential mechanisms of early-life PM_2.5_ exposure and health effects in adulthood. Epidemiological studies indicated that prenatal environmental conditions resulted in epigenetic changes which persist the whole life (Heijmans et al. 2008). Evidence from animal studies indicated that certain environmental conditions in utero could cause persistent epigenetic dysregulation, resulting in life-long consequences (Anway et al. 2005). Both the global and gene-specific methylation could be affected by particulate matter (Baccarelli et al. 2009; Madrigano et al. 2011).

In our present study, the real-world PM_2.5_ exposure system was used to explore the long-term effects of maternal exposure to concentrated ambient PM_2.5_ on male mice offspring spatial learning and memory competence. Furthermore, the hypothesis of potential mechanisms was that methylation modification plays an important role in the effects of prenatal PM_2.5_ exposure on spatial memory in mice offspring.

## Methods

### Animal exposure to PM_2.5_

**Fig. 1:**
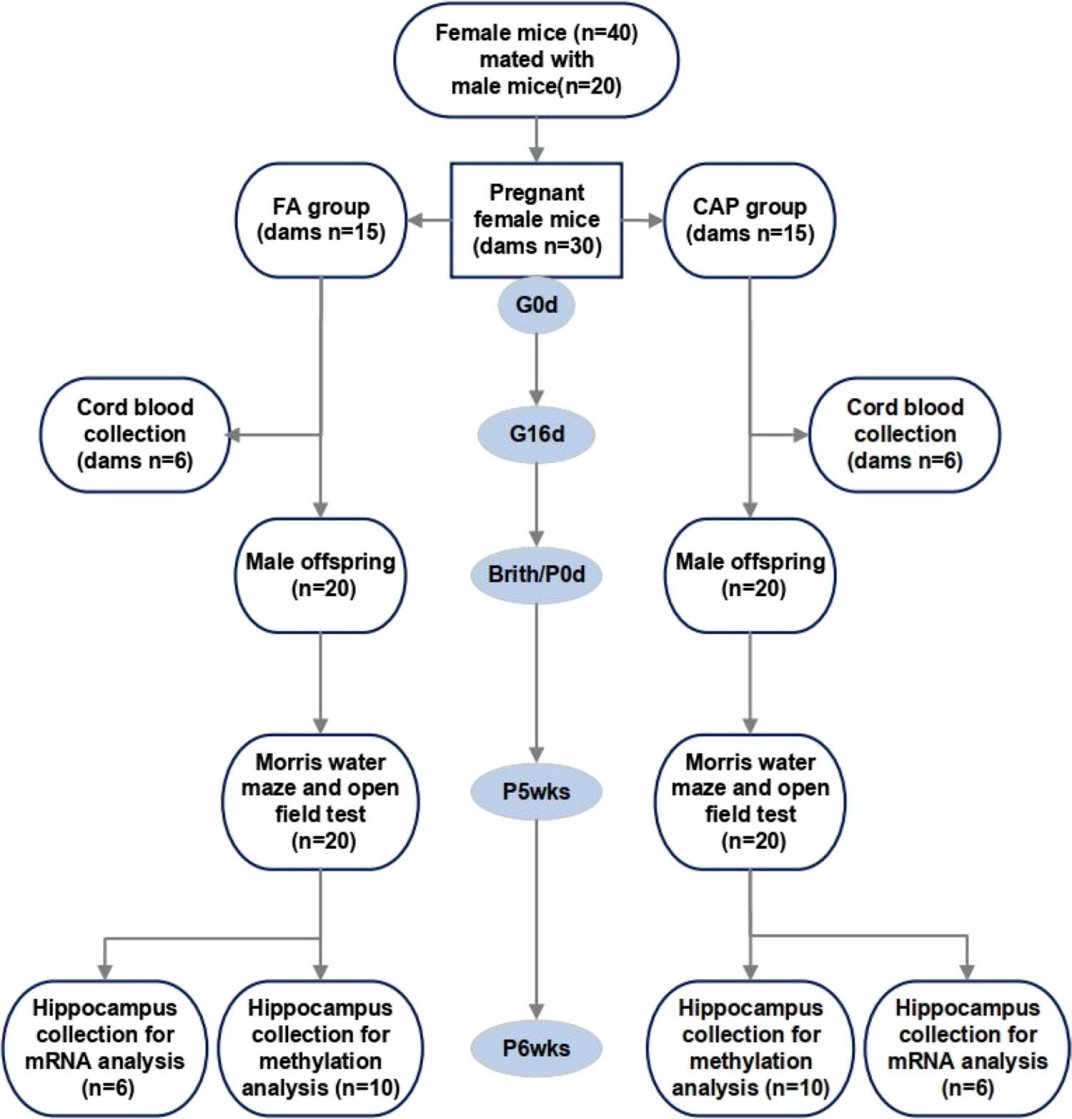
The summary of the study design. Abbreviations: FA, filtered air; CAP, concentrated ambient PM_2.5_. G0d: Gestational 0 day; G16d: Gestational 16 day; P0: postnatal 0 day; P5wks: postnatal 5 weeks; P6wks: postnatal 6 weeks

The study design is summarized in Fig. 1. A total of 40 female and 20 male 6-week-old C57BL/6 mice were obtained from Shanghai Lingchang Biotech Limited Company (certification No. 2013001821608). After a 1-week adaptation period, every two female mice were mated with one male mouse per cage. Seminal plugs were then checked 12–18 h after the breeding pairs were cohoused. Gestation day 0 (GD0) was defined as the day on which upon the appearance of a vaginal plug. There was a total of 30 plug-positive mice which were considered pregnant. The pregnant mice were divided into a concentrated ambient PM_2.5_ group (CAP) (*n* = 15) and a filtered air group (FA) (*n* = 15) randomly.

Animals were respectively exposed to filtered air or concentrated ambient PM_2.5_ in “Shanghai Meteorological and Environmental Animal Exposure System, Shanghai-METAS” (patent #2015104536008-) (Du et al. 2018; Yang et al. 2019) from GD0 to the delivery day. The “Shanghai-METAS” is located in the center of Shanghai city, and most of the PM_2.5_ components are attributed to traffic exhausts. The concentrated PM_2.5_ in the exposure chamber was generated using a versatile aerosol concentration enrichment system, while the air in the control chamber was filtered by a high-efficiency particulate-air filter which removed 98% of ambient particles (Pan et al. 2021; Sun et al. 2005; Maciejczyk et al. 2005). In the living environment of “Shanghai-METAS,” the light circle was 12 h light/12 h dark, the temperature was18–25 °C, and the relative humidity was 40–60%. Food and water were provided ad libitum. The duration of the perinatal exposure was 8 h per day, 7 days per week for about 18 days. The average PM_2.5_ concentrations in the FA and CAP chambers during the exposure period were (2.78 ± 1.19) μg/m^3^ and (102.99 ± 78.74) μg/m^3^, respectively. The average ambient PM_2.5_ concentration during the exposure period was (36.57 ± 17.46) μg/m^3^.

In both of CAP and FA groups, the number of dams was 15. Among them, 6 dams from each group were randomly selected and sacrificed for cord blood collection at gestational 16 days. The other 9 dams gave birth 20 male offspring in each group. After giving birth, the maternal mice and pubs were raised in the SPF animal room for 3 weeks, when the pubs were weaned and redistributed by gender. All the male offspring were conducted for the Morris Water Maze test and open field test at postnatal 5 weeks. When finished with the behavior tests, the mice offspring were sacrificed for hippocampus collection. Among them, 10 hippocampus tissues from each group were selected randomly for methylation analysis, and 6 hippocampus tissues were selected randomly from each group for mRNA analysis. The investigators who conducted the Morris Water Maze test, open field test, and tissue samples analysis were all blinded to the group allocation.

The Animal Experimental Ethics Committee of the Department of Laboratory Animal Science, Fudan University, approved all procedures of this study, with an ethics reference number 201805003Z. All animals were treated humanely and with regard for the alleviation of suffering.

### Morris water maze test

Spatial learning and memory ability were assessed using the Morris water maze test when the offspring mice were 5 weeks old. The Morris Water Maze device was a cylindrical tank (120 cm in diameter and 50 cm in depth). The pool was filled up with tap water of 30 cm in depth, which was colored opaque with powdered non-fat milk or non-toxic tempera paint. The device was artificially divided into four quadrants (Northwest (NW), Northeast (NE), Southeast (SE), Southwest (SW)), using four points on the rim of the pool, North (N), South (S), East (E), and West (W) (not true magnetic directions). The escape platform (6 cm in diameter) was placed 1 cm below the surface of the water at the southwest quadrants (target quadrant). In the acquisition test phase, each mouse was trained in four trials per day for five consecutive days. In each trial, the mouse was gently placed into the pool facing the wall at one of the four quadrants, randomly. The trial was completed when the mouse swam to and climbed onto the hidden goal platform. If the mouse failed to locate the platform within 60 s, it was guided to the platform by a stick. The mice were left to stay on the platform for 30 s, before they were returned to the cages. The time to reach the platform was recorded as escape latency. The probe test was conducted following the acquisition test. After all the mice had completed the acquisition trials, the platform was removed for probe test 24 h after the last acquisition day. In the probe test, the mouse was released from the opposite quadrant of the target quadrant and was allowed to swim freely for 1 min. The swimming distance within the target quadrant, the time spent in the target quadrant, and the number of target quadrant entries were recorded during the 60 s by the ANY-Maze video tracking system (Stoelting Co., U.S.A). All tracks from all trials were analyzed for several behavioral parameters using SMART software (Panlab).

### Open field test

The open field test was applied to assess the offspring’s ability to cope with escapable stressful situations (Laugeray et al. 2018). To let the mice acclimate to the experimental room, the mice were transported to the test room and left undisturbed for 30 min before the test. The open field was set up according to the experimental manufacturer. Each mouse was placed in a corner of the open field individually and left for 10 min to explore the entire apparatus. The EthoVision video-tracking system (Noldus, The Netherlands) was applied to record and analyze the activities. The travel variables, including the total travelled distance, the time spent in the center, in the intermediary, and the intermediary in the arena as well as the mean speed in each zone, were automatically recorded. We wiped the apparatus clean using a 50% ethanol/water solution and allow time for it to dry between mice.

The mice were sacrificed when both the Morris Water Maze test and open field test were done. The hippocampus was harvested to conduct DNA methylation analysis (10 samples per group) and mRNA analysis (6 samples per group).

### Analysis of the DNA methylation status

The genomic DNA of the hippocampus was extracted using the TIANamp Genomic DNA Kit (TIANGEN BIOTECH, China). Genomic DNA bisulfite conversion was performed using the EpiTeck Bisulfite Kit (QIANGEN), which was ready for bisulfite sequencing PCR (BSP) analysis. The primers of IL-6, IL-1β, TNF-α, and BDNF were designed using PyroMark Assay Design 2.0 (BGI, China). The target promoter sequences of IL-6, IL-1β, TNF- α, and BDNF were amplified from the converted DNA performed through touch-down PCR, and then the purified PCR products were cloned into pMD-18 T vector (Takara, China) for sequencing. Pyrosequencing was performed using PyroMark Q96 ID software (QIAGEN), and the CpG Island level was analyzed automatically.

### Quantitative real-time PCR

Total RNA was extracted from the hippocampus using TRIzol® reagent (Invitrogen, USA) according to the manufacturer’s instruction. The RNA was then reverse-transcribed into cDNA using the PrimeScript™ RT reagent kit (Takara Bio, Inc.), at 37 ℃ for 15 min and 80℃ for 5 s. PCR amplification was performed on a StepOnePlus™ real-time PCR detection system using TB Green™ Premix Ex Taq™ II (Tli RNaseH Plus) (cat. no. RR820A, Takara Bio, Inc.). The following conditions were used for PCR amplification: initial denaturation at 95℃ for 30 s, followed by 40 cycles of denaturation at 95 ℃ for 30 s, annealing at 60 ℃ for 30 s. The primer sequences for IL-6, interleukin 1β (IL-1β), tumor necrosis factor-a (TNF-a), brain-derived neurotrophic factor (BDNF), and β-actin shown in Table 1 were designed and synthesized by primer Express Software (Beijing Genomic Institute, China. Relative changes of mRNA expression were analyzed using the 2^−△△Ct^ method (Livak and Schmittgen 2001) and normalized to the internal reference gene GAPDH. All experiments were performed in triplicate.

**Table 1:** Primer sequences for quantitative real-time PCR

Primer name	Base sequence (5 to 3′)	TM
IL6-F	CACAAGTCCGGAGAGGAGAC	60
IL6-R	TCCACGATTTCCCAGAGAAC	
IL1β-F	ATCTCGCAGCAGCACATCAA	60
IL1β-R	CTCATCCTGGAAGGTCCACG	
TNF-F	CGGCATGGATCTCAAAGACA	60
TNF-R	ATAGCAAATCGGCTGACGGT	
BDNF-F	CCAAAGGCCAACTGAAGCAG	60
BDNF-R	TGCGAGTTCCAGTGCCTTTT	
mACTIN-F	ACTTCGAGCAGGAGATGGCC	60
mACTIN-R	CCCAAGAAGGAAGGCTGGAA	

### Statistical analysis

Continuous variables were reported as the mean ± standard error (SE) when met normal distribution; otherwise, the median and interquartile range were reported. The Kolmogorov–Smirnov test was applied to determine the normal distribution. The differences between two groups of body weight, tissue weight, metal concentration, open field test data, mRNA, and DNA methylation data were compared using a two-tailed students’ *t*-test. For the data of probe trials in Morris Water Maze were not normally distributed, Mann–Whitney *U* test by rank was used to compare the differences between groups. The repeated measurement of 5-day average escape latency in the acquisition trials of Morris Water Maze was analyzed using two-way repeated measures ANOVA with Bonferroni post-hoc test (Vorhees and Williams 2006). Furthermore, the data of acquisition trials in Morris Water Maze at each time point did not fit a normal distribution; the differences between groups were evaluated using Mann–Whitney *U* test by rank. Data statistical analyses were conducted using SPSS software (SPSS program, version 26.0, Chicago, IL, USA). A *p*-value less than 0.05 was considered statistically significant. The graphs were generated using GraphPad Prism software for Windows (Version 9.2.0, GraphPad Software, Inc).

## Results

### PM_2.5_ exposure concentration

PM_2.5_ exposure concentrations were estimated by averaging particle weights from multiple Teflon filters across the treatment period. The average PM_2.5_ concentrations in the FA and CAP chambers were comparable. CAP filters contained almost 43-fold increase in the concentration of particulates (102.99 ± 78.74) μg/m^3^ compared to FA filters (2.78 ± 1.19) μg/m^3^and 2.82-fold the concentration found in the ambient air (36.57 ± 17.46) μg/m^3^. Filters from ambient PM_2.5_ were collected and further analyzed for particle components including carbons, elements, and ions. Detailed analysis results of particle components can be found in the supplementary materials.

### Pups body weight, tissue weight, lead and mercury concentrations of dams’ cord blood

As shown in Table 2, maternal exposure to CAP did not significantly alter the mice offspring’s body weight. Significant differences in the weights of offspring’s hippocampus and prefrontal cortex between the CAP and FA groups were not observed. The concentrations of lead and mercury in the dams’ blood were tested. The mercury concentration of the dams’ cord blood in the CAP group (4.77 ± 0.84) μg/kg was higher than that in the FA group (2.63 ± 0.15) μg/kg, with statistical significance (*p* = 0.030). The lead concentration of the dams’ cord blood in the CAP group was (4.69 ± 0.61) μg/L and (4.47 ± 0.81) μg/L in the FA group, and the difference was not statistically significant (*p* = 0.837).

**Table 2:** Pub body weight and tissue weight (*n* = 20 per group, mean ± SE)

	FA	CAP	*p*-value
Body weight (g)	23.37 ± 0.30	23.11 ± 0.25	0.518
Weight of hippocampus (mg)	27.95 ± 2.18	29.14 ± 2.18	0.701
Weight of prefrontal cortex (mg)	21.28 ± 2.64	22.71 ± 3.18	0.731

Abbreviations: *FA*, Filtered air; *CAP*, concentrated ambient PM_2.5_

### Maternal exposure to CAP impaired the spatial memory ability in male mice offspring

**Fig. 2:**
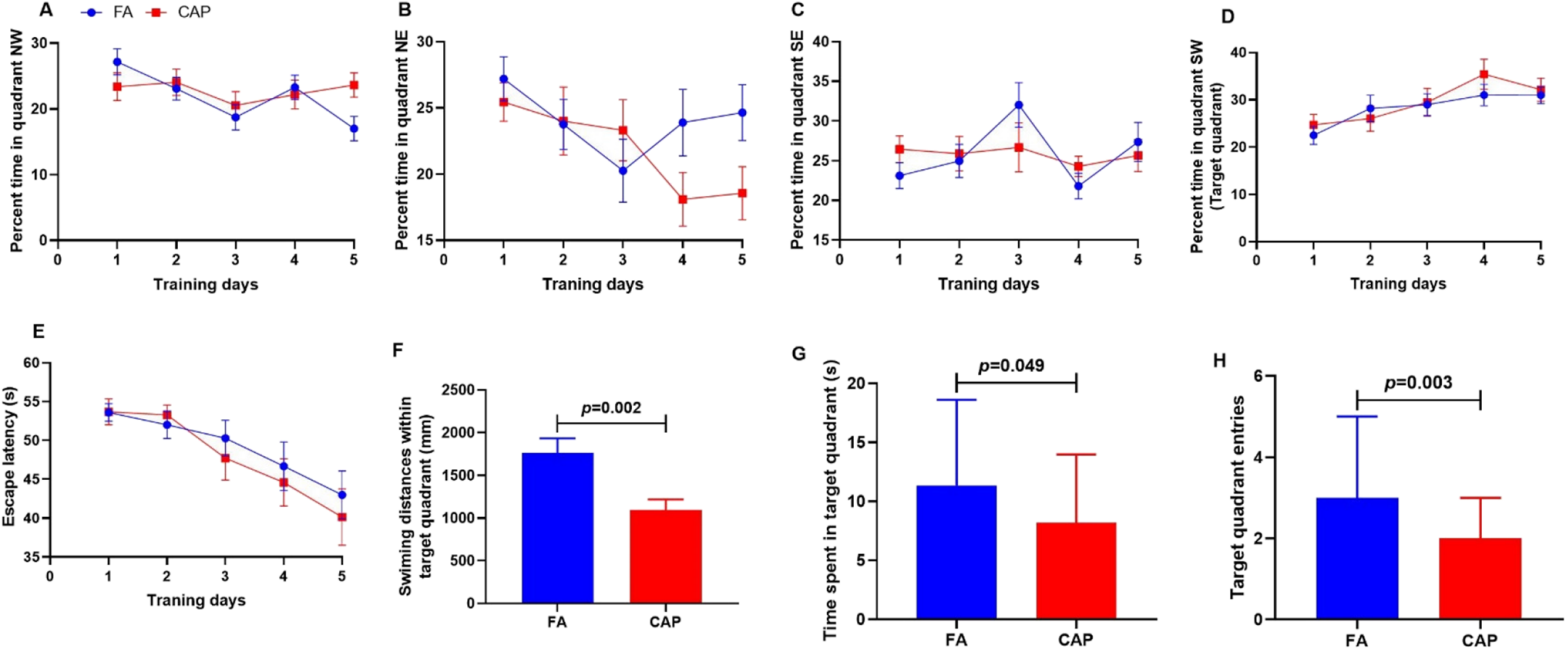
Maternal concentrated ambient PM_2.5_ exposure impaired the spatial memory ability in male mice offspring tested by Morris Water maze. **A**–**D** Percent time in each quadrant of Morris water maze performance on each day during the acquisition trials. **A** Quadrant NW, **B** Quadrant NE, **C** Quadrant SE, **D** Quadrant SW). **E** Escape latency during the training days; **F** Swimming distances within the target quadrant; **G** time spent in the target quadrant; **H** number of entries in the target quadrant. (*n* = 20 per group). Abbreviations: FA, filtered air; CAP, concentrated ambient PM_2.5_. NW, Northwest; NE, Northeast; SE, Southeast; SW, Southwest

Spatial learning and memory ability of mice offspring was evaluated by performing the Morris water maze. The percentage time in each quadrant of the Morris water maze performance on each day during the acquisition trials is shown in Fig. 2A–D. Increasing trends of percentage time were observed in the target quadrant (quadrant SW, Fig. 2D) in both groups, suggesting the mice offspring had the ability of discriminating between the target quadrant and all other quadrants. Changes in escape latency time in 5-day acquisition trials are shown in Fig. 2E. Analysis of two-way repeated measures ANOVA showed that there was no significant interaction between groups and days (interaction effects: *F*(4, 76) = 0.272, *p* = 0.895). The escape latency of training mice decreased as training days increased (time main effect: *F*(4, 76) = 7.956, *p* < 0.001), indicating that mice in each group had learning abilities. The differences of the escape latency between the two groups were not statistically significant (group main effect: *F*(1, 19) = 0.663, *p* = 0.426). Specifically, the results of Mann–Whitney *U* test showed that the escape latency on each day of acquisition trials did not differ between the groups.

The results from the probe test of Morris Water Maze showed that, compared with the FA group, the swimming distance within the target quadrant was significantly shorter in the CAP group *(p* < 0.05) (Fig. 2F). In addition, the time spent in the target quadrant and the number of target quadrant entries in CAP group were significantly less than FA group (*p* < 0.05) (Fig. 2G, H). Collectively, the results showed that maternal exposure to high concentration PM_2.5_ impaired the spatial memory ability in mice offspring.

### Maternal exposure to CAP did not differ the anxiety-like behavior in male mice offspring

**Fig. 3:**
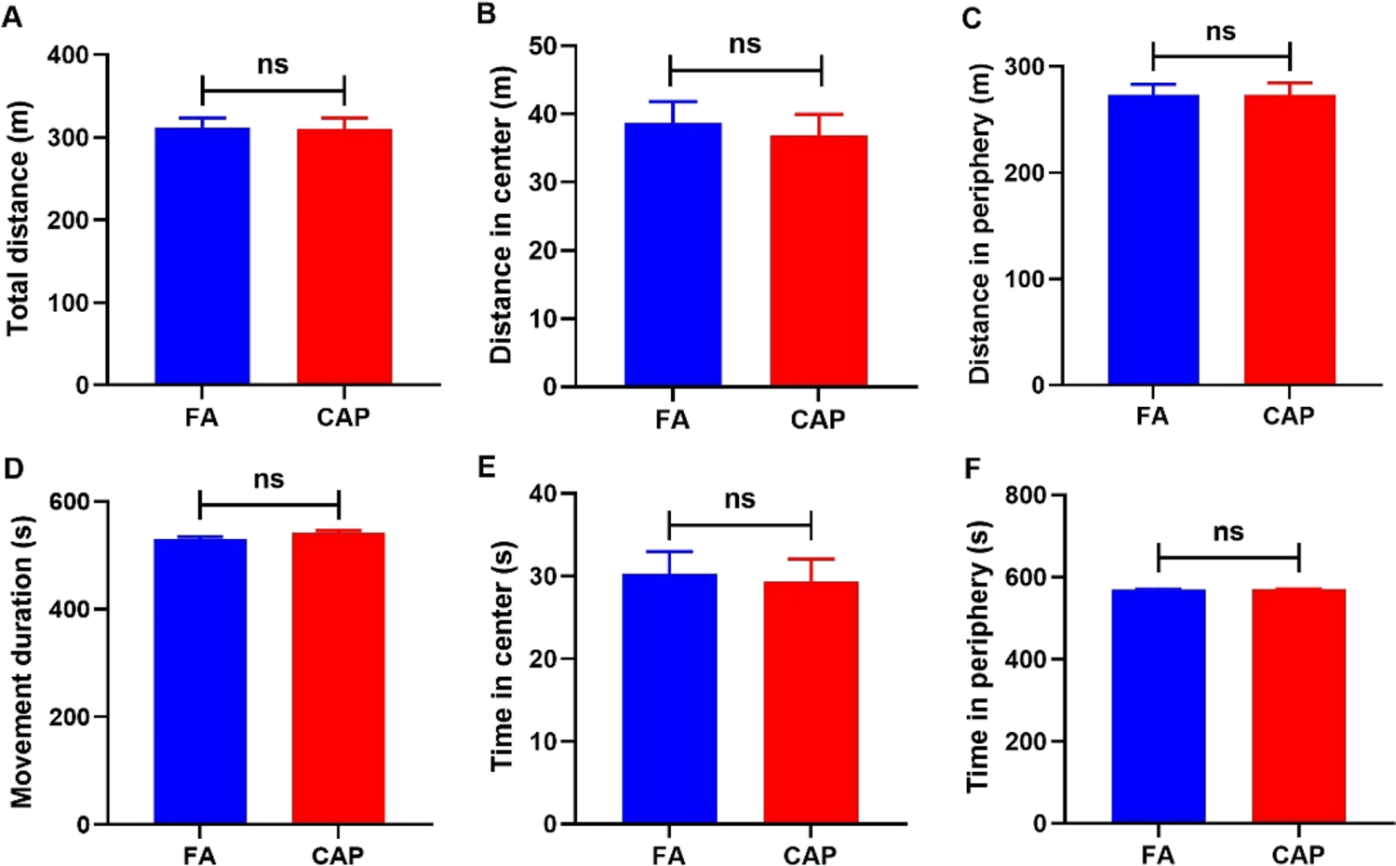
Maternal concentrated ambient PM_2.5_ exposure did not impact anxiety-like activity in male mice offspring. **A** Total distance travelled; **B** distance travelled in central area; **C** distance travelled in periphery area; **D** movement duration; **E** duration stayed in central area; **F** duration stayed in periphery (*n* = 20 per group). Abbreviations: FA, filtered air; CAP, concentrated ambient PM_2.5_

The open field test was conducted to assess whether maternal exposure to concentrated ambient PM_2.5_ altered the spontaneous locomotor activity and exploratory behavior in mice offspring. No statistically significant differences were detected in total distance travelled (Fig. 3A), total movement duration (Fig. 3D), distance travelled, and duration in the periphery area (Fig. 3C, F) between the two groups of mice offspring. Although the CAP group offspring showed shorter travelling distance and duration in the central area than the FA group (Fig. 3B, E), the differences between the two groups were not statistically significant. The results of the open field test suggested that anxiety-like behavior in mice offspring did not differ.

### mRNA expression levels of inflammation related cytokines in mice offspring hippocampus

**Fig. 4:**
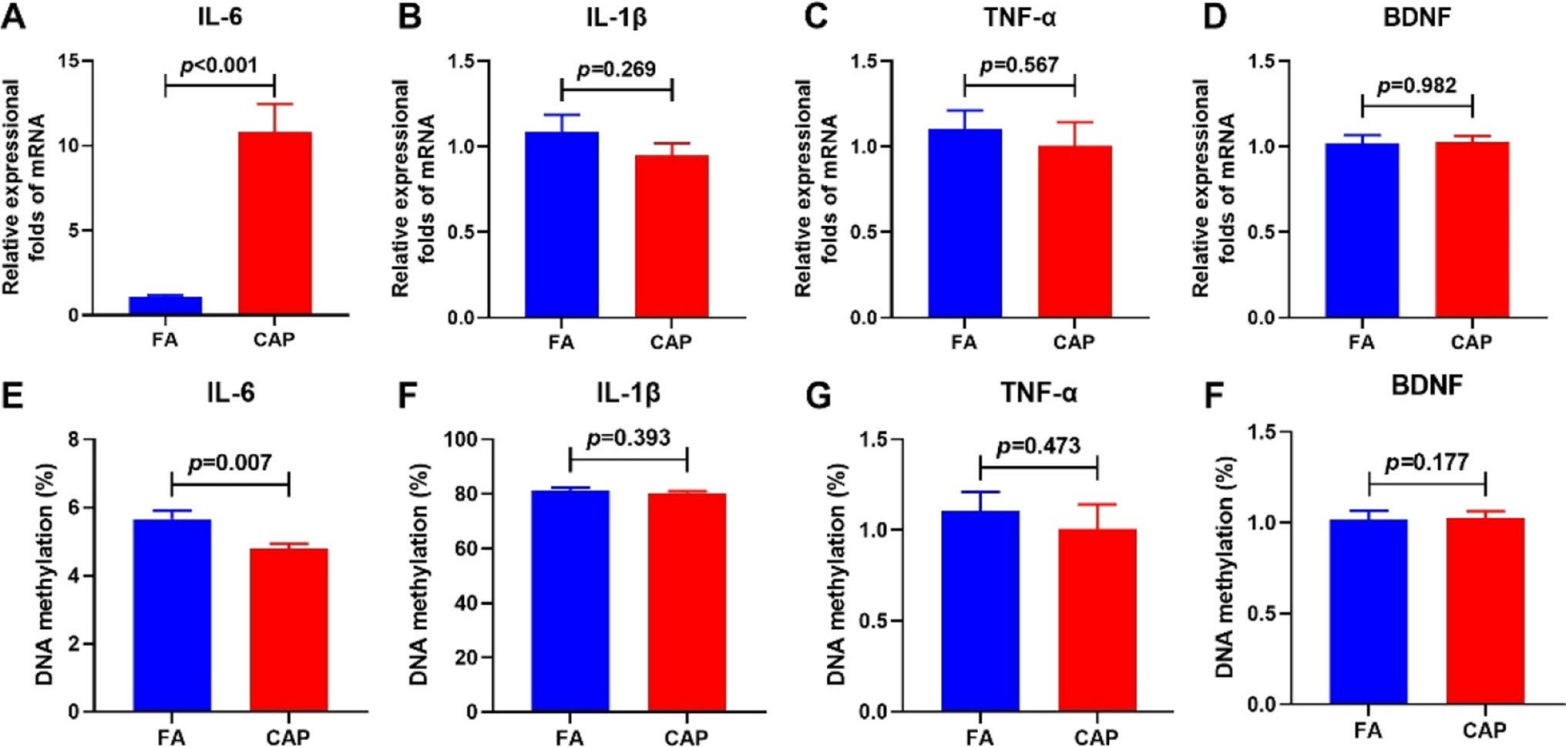
The expressions of inflammation-related mRNA (**A**–**D**) and DNA methylation modification (**E**–**H**) in the hippocampus. The data are expressed as the (mean ± SE). *N* = 6 per group for mRNA analysis and three replicates were conducted for each sample. *N* = 10 per group for DNA methylation analysis. Abbreviations: FA, filtered air; CAP, concentrated ambient PM_2.5_; SE, standard error. Students’ t-test were used to test the difference between the two groups

To investigate the effects of PM_2.5_ exposure on the expression levels of IL-6, IL-1β, TNF-α, and BDNF in the hippocampus, quantitative real-time PCR was performed. The mRNA level of IL-6 in the CAP group (10.80 ± 7.03) increased significantly in the hippocampus than that in the FA group (1.08 ± 0.43) (Fig. 4A). We did not observe a significant difference in mRNA levels of IL-1β, TNF-α, and BDNF between the CAP and FA groups (Fig. 4B, C, D).

### Analysis of DNA methylation modification

The effects of PM_2.5_ exposure during pregnancy on the methylation modification in the IL-6, IL-1β, TNF-α, and BDNF promoter regions in the hippocampus were investigated using bisulfite sequencing PCR. As shown in Fig. 4E, compared with the FA group (5.66 ± 0.83) %, the methylation levels of the CpG sites in the IL-6 promoter region declined significantly in the CAP group, (4.79 ± 0.48) %. The differences in the methylation levels of IL-1β, TNF-α, and BDNF between the two groups were not significant (Fig. 4F, G, H).

## Discussion

Increasing evidence demonstrated that concentrated ambient particulate matter exposure affected the behavioral, neurochemical, and neuropathological development (Allen et al. 2013, 2017b). The relationship between PM_2.5_ exposure and neurodevelopment has become a great public health concern in recent decades. The conclusions of existing epidemiology studies from different populations and animal models were somewhat inconsistent, which might be owing to the discrepancy in particle components, particle size, exposure duration, and exposure level in the various studies (Kim et al. 2015). The neurotoxicological mechanisms of air pollution impairment had not yet been identified, for it was likely to differ depending on the specific nature of the cognitive impairment, the developmental period of exposure, the components of pollution, and even sex (Allen et al. 2017a). Notably, studies suggested that prenatal air pollution exposure showed sex-specific neuropsychological effects in offspring (Lertxundi et al. 2019; Bolton et al. 2017), which specifically caused stronger effects on males. In particular, we focused on the PM_2.5_ exposure effects on male offspring.

In the current study, concentrated PM_2.5_ exposure during pregnancy reduced the male mice offspring spatial memory ability assessed by the Morris water maze test. Our results were consistent with previous reports. Studies reported that neonatal rat exposure to PM_2.5_ through intranasal instillation impaired spatial learning and memory abilities led to increased anxiety-like symptoms and apparent depressive-like behaviors (Liu et al. 2019). Gestational PM_2.5_ exposure led to neurobehavioral defects including anxiety- and depression-like behavior, tested by the open field test and tail suspension test (Wang et al. 2020). An epidemiological study reported that prenatal exposure to ambient particulate matter was negatively associated with children’s neurodevelopment throughout the first 24 months of life (Kim et al. 2014). However, Hougaard et al. (2008). found that the cognitive function and levels of biomarkers of prenatal exposure to diesel exhaust particles (DEP) were generally similar in exposed and control offspring. The inconsistent results might be due to the composition of the particulates and exposure methods. Meanwhile, it should be noted that most of the PM_2.5_ components are attributed to traffic exhausts; therefore, PM_2.5_ from other sources may not necessarily lead to similar results from this study.

The developing central nervous system was especially susceptible to the toxic effects of metals and metal components, including mercury and lead. Epidemiological studies demonstrated that low levels of prenatal Hg exposure may cause early childhood neurocognitive effects (Karagas et al. 2012). There was convincing experimental evidence that exposure to low levels of neurotoxic substances during vulnerable developmental periods can induce permanent functional disturbances in the central nervous system (Andersen et al. 2000). We found a higher mercury concentration in the dams’ cord blood in the concentrated PM_2.5_ group. Collectively, the metals consisted in PM_2.5_ might play an important role in the negative effect on spatial learning of prenatal PM_2.5_ exposure.

Growing evidence suggested that air pollutants may adversely affect the central nervous system. Peripheral inflammation itself can adversely affect the central nervous system, thus direct translocation of particle pollutants into the brain would not be necessary to elicit neurotoxicity (Allen et al. 2013). Acutely exposed to diesel exhaust showed microglia activation, increased lipid peroxidation, and neuro-inflammation in various mice brain regions, particularly the hippocampus and the olfactory bulb (Costa et al. 2017). Air pollution exposure–induced neuroinflammation might be one of the potential mechanisms of exposure-induced adverse outcomes on the nervous system (Calderon-Garciduenas et al. 2015). In the current study, gestational exposure to concentrated PM_2.5_ elevated the mRNA expression of the inflammatory cytokine IL-6 in the hippocampus of offspring mice. Significant differences in IL-1β, TNF-α, and BDNF mRNA expression between the two groups were not observed. Zheng et al. (2018) also demonstrated that gestational exposure to PM_2.5_ was associated with increased secretions of inflammatory proteins (including NF-κB, TNF-α, IL-1β) with a dose–response relationship. A clinical study reported that PM_2.5_ exposure significantly increased the concentration of IL-6 and TNF-α compared to baseline, while without a significant decrease of BDNF concentration in the sera of adults (Cliff et al. 2016). However, molecular epidemiological evidence from a mother-infant pairs cohort study reported that PM_2.5_ exposure in utero was negatively associated with the placental expression of BDNF at birth (Saenen et al. 2015). PM_2.5_ exposure after birth decreased BDNF expression level of BDNF in the hippocampus. These inconsistent results between different studies may be ascribed to differences in neurodevelopmental stage, PM_2.5_ exposure duration, and the specimen sample. In the central nervous system, IL-6 is an important signaling molecule. IL-6 is produced by resident cells in the brain. Under normal conditions, the expression of IL-6 in the central nervous system is generally low (Gruol 2015). The increasing expression of IL-6 was involved in the deterioration of cognitive functions during aging and neurodegenerative diseases (Weaver et al. 2002). Although the current study could not provide direct evidence of a causal relationship between elevated IL-6 expression and spatial memory impairment, the causal relationship could be supported by previous studies. IL-6 over-expression mice in the central nervous system exhibited inflammatory neurodegeneration and learning impairment (Heyser et al. 1997). Moreover, IL-6 deficiency mice (IL-6 knockout) presented better spatial reference memory, slower age-related memory decline (Bialuk et al. 2018), and improvement in long-term memory (Bialuk and Winnicka 2018).

Epigenetic regulation of gene transcription, including DNA methylation, is sensitive to environmental pollution. Environmental pollution not only affects the total methylation modification but also affects the methylation of specific genes. Gestational exposure to air pollutants can lead to locus-specific changes in gene methylation, which is involved in cellular responses to inflammation and early-life development, in newborn cord blood and placenta (Isaevska et al. 2021). In the present study, high-level PM_2.5_ exposure during pregnancy significantly elevated the mRNA levels of IL-6 in the hippocampus tissue. Interestingly, PM_2.5_ exposure during pregnancy induced lower methylation levels of the CpG sites in the IL-6 promoter region. Previous evidence suggested that methylation modification was an essential epigenetic mechanism regulating IL-6 expression (Poplutz et al. 2014), and the DNA methylation levels of the CpG islands in the IL-6 promoters were inversely significantly correlated to the corresponding mRNA expression (Tekpli et al. 2013). In addition, increasing studies demonstrated that epigenetic mechanisms, including DNA methylation, play important roles in neurogenesis (Wang et al. 2016). Our results suggested that the hypomethylation level in the IL-6 gene was associated with higher mRNA levels of IL-6 proteins in the hippocampus, consequently resulting in spatial memory impairment.

To our knowledge, the current study was the first one to explore the epigenetic mechanism of DNA methylation, for the male offspring memory impairment induced by prenatal particulate matter exposure. Notably, a whole-body inhalational system, “Shanghai METAS,” was used to establish the animal exposure model. As the key part of “Shanghai METAS,” the versatile aerosol concentration enrichment system (VACES) had been modified, assembled, tested, and validated by researchers (Maciejczyk et al. 2005). Compared with the intratracheal instillation exposure way, the “Shanghai METAS” can mimic human’s real-world exposure to the environmentally relevant PM_2.5_ or filtered air to the maximum extent (Pan et al. 2021; Yang et al. 2019; Ying et al. 2014).

Despite the novelty of the findings, there were still several limitations in this study. Although the PM_2.5_ exposure duration might be considered real-world, the concentrations used were not likely real-world. The current experiment did not expose the dams with PM_2.5_ of ambient concentrations, which may constitute a more reasonable exposure gradient and reflect real-world exposure scenarios. The PM_2.5_ concentration in concentrated PM_2.5_ exposure was higher than the clinic and epidemiology reports, which might not reflect the effects on spatial memory of conventional PM_2.5_ concentration in the atmospheric environment. Therefore, the results should be interpreted with certain environmental implications, such as pregnant women living in heavily polluted regions. Neural development was a developmental process and the effects of particulate matter exposure during pregnancy on the memory ability of offspring at different growth stages were not measured. Morris Water maze was used to assess the spatial learning and memory ability. However, the minor time spent in the target quadrant in the probe test of Morris Water Maze performance could be attributed to less persevering behavior or perhaps an adaptive advantage. Although the compositions of the ambient particulate matter were measured, it was not possible to further analyze the specific components that caused the impairment of the offspring’s memory ability. Instead of measuring the global methylation, selected genes were methylated based on previous studies and hypotheses. The inflammatory factors and BDNF were tested only in the hippocampus but other brain regions probably involve in the memory deficits, such as the prefrontal cortex. Although we found the changes in mRNA and DNA methylation of IL-6, the results could not provide direct evidence for a causal relationship between these and the spatial memory impairment. Finally, we did not explore the neurodevelopment effects on female mice offspring of gestational PM_2.5_ exposure. Further studies will be warranted to illustrate these problems in the future.

## Conclusion

Together, our results demonstrated that maternal exposure to concentrated ambient PM_2.5_ induced long-lasting spatial memory development defects in male mice offspring. The underlying biological mechanism might be mediated by the inflammatory reaction which is regulated by DNA methylation. Future studies are needed to explore the effects of high PM_2.5_ exposure during pregnancy on offspring spatial memory ability and to clarify the biological mechanisms involved.

## Supplementary Information

Below is the link to the electronic supplementary material.

Supplementary file1 (DOCX 94 KB)

## Data Availability

The data supporting the conclusions of this article are available from the corresponding author upon reasonable request.
