# Supplementary material for: Prenatal exposure to concentrated ambient PM2.5 results in spatial memory defects regulated by DNA methylation in male mice offspring
Source: Environ Sci Pollut Res Int. 2022 Dec 17;30(12):35142–52. doi: 10.1007/s11356-022-24663-5 (PMC10017658; doi:10.1007/s11356-022-24663-5)

**Supplementary materials**

**Article Title:** Prenatal exposure to concentrated ambient PM_2.5_ results in spatial memory defects regulated by DNA methylation in male mice offspring

Filters from ambient PM_2.5_ were collected and further analyzed for particle components including carbons, elements and ions. The concentrations of elemental carbon (EC) and organic carbon (OC) were 6.4 μg/m^3^ and 6.0 μg/m^3^ respectively. As shown in Figure S1, the most abundant metal constituents were Na (37.76%), Zn (32.89%), Ba (13.84%), K (7.21%) and Ca (2.52%), while the most abundant ions were SO42- (40.07%), NO3- (31.15%), Na+ (13.80%), NH4+ (7.41%) and K+ (3.45%).

**Figure S1. Percentages of the Components in the concentrated ambient PM_2.5_**


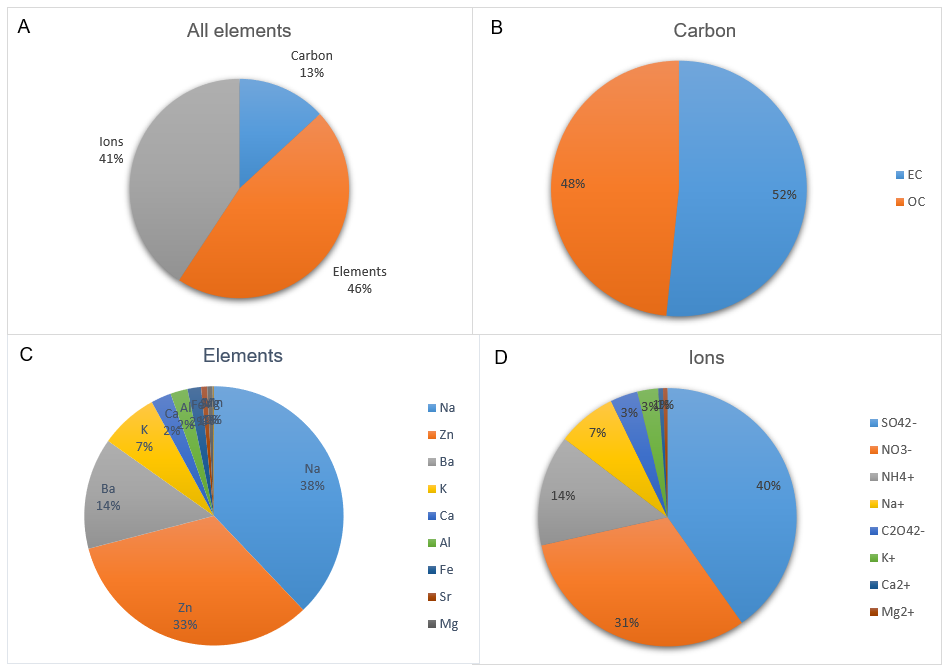

Supplement: Supplementary file 1 — Supplementary file1 (DOCX 94 KB) [file 11356_2022_24663_MOESM1_ESM.docx]
